# Exploring the impact of indication on variation in rates of intrapartum caesarean section in six Palestinian hospitals: a prospective cohort study

**DOI:** 10.1186/s12884-022-05196-8

**Published:** 2022-12-02

**Authors:** Mohammed W. Zimmo, Katariina Laine, Sahar Hassan, Bettina Bottcher, Erik Fosse, Hadil Ali-Masri, Khaled Zimmo, Ragnhild Sørum Falk, Marit Lieng, Ase Vikanes

**Affiliations:** 1 Faculty of Medicine, Institute for Clinical Medicine, University of Oslo, Oslo, Norway; 2 The Intervention Centre, Oslo University Hospital, Oslo, Norway; 3 Department of Obstetrics and Gynecology, Al Shifa Hospitals, Gaza, Palestine; 4 Institute for Clinical Medicine, University of Oslo, Oslo, Norway; 5 Norwegian Research Centre for Women’s Health, Oslo University Hospital, Oslo, Norway; 6 Faculty of Nursing, Pharmacy and Health Professions and Institute of Community and Public Health, Birzeit University, Ramallah, Palestine; 7 Faculty of Medicine, Islamic University of Gaza, Gaza, Palestine; 8 Intervention Centre, Oslo University Hospital Rikshospitalet, Oslo, Norway; 9 Obstetrics Department, Palestine Medical complex, Ramallah, Palestine; 10 Department of Obstetrics and Gynecology, Al Aqsa Hospital, Gaza, Palestine; 11 Oslo Centre for Biostatistics and Epidemiology, Research Support Services, Oslo University Hospital, Oslo, Norway; 12 Institute for Clinical Medicine, Faculty of Medicine, University of Oslo, Oslo, Norway; 13 Department of Gynecology, Oslo University Hospital, Oslo, Norway

**Keywords:** Caesarean section, Intrapartum, Gaza, Indications, Odds, Palestine, West Bank

## Abstract

**Background:**

Caesarean section rates are rising globally. No specific caesarian section rate at either country-level or hospital-level was recommended. In Palestinian government hospitals, nearly one-fourth of all births were caesarean sections, ranging from 14.5 to 35.6%. Our aim was to assess whether variation in odds for intrapartum caesarean section in six Palestinian government hospitals can be explained by differences in indications.

**Methods:**

Data on maternal and fetal health were collected prospectively for all women scheduled for vaginal delivery during the period from 1st March 2015 to 30th November 2016 in six government hospitals in Palestine. Comparisons of proportions in sociodemographic, antenatal obstetric characteristics and indications by the hospital were tested by χ2 test and differences in means by one-way ANOVA analysis. The odds for intrapartum caesarean section were estimated by logistic regression. The amount of explained variance was estimated by Nagelkerke R square.

**Results:**

Out of 51,041 women, 4724 (9.3%) underwent intrapartum caesarean section. The prevalence of intrapartum caesarean section varied across hospitals; from 7.6 to 22.1% in nulliparous, and from 5.8 to 14.1% among parous women. The most common indications were fetal distress and failure to progress in nulliparous, and previous caesarean section with an additional obstetric indication among parous women. Adjusted ORs for intrapartum caesarean section among nulliparous women ranged from 0.42 (95% CI 0.31 to 0.57) to 2.41 (95% CI 1.70 to 3.40) compared to the reference hospital, and from 0.50 (95% CI 0.40–0.63) to 2.07 (95% CI 1.61 to 2.67) among parous women. Indications explained 58 and 66% of the variation in intrapartum caesarean section among nulliparous and parous women, respectively.

**Conclusion:**

The differences in odds for intrapartum caesarean section among hospitals could not be fully explained by differences in indications. Further investigations on provider related factors as well as maternal and fetal outcomes in different hospitals are necessary.

## Background

Worldwide, caesarean section rates are rising [1]. On one hand, the caesarean section rates among healthy nulliparous women with singleton pregnancies at term, who have a low risk of caesarean section, have been constantly rising [2]. On the other hand, there is a need for better caesarean section availability, particularly in low and middle income countries, which is an essential component of comprehensive emergency obstetric and neonatal care (CEmONC) [3]. In the WHO report on the caesarean section from 2015, no specific caesarian section rate at either country-level or hospital-level was recommended [4]. However, caesarean section was recommended only to be performed with an appropriate indication [4].

In Palestine, obstetric care and delivery services are offered in government as well as private maternity hospitals. Government hospitals are available in all geographic areas and offer services at very low costs [5]. In Gaza, 77.4% of births take place in government hospitals, compared to 51.2% in the West Bank [5, 6]. One-to-one care, which is an important intervention to prevent caesarean section, is not available in labour wards of government hospitals in Palestine [6]. In 2015 nearly one-fourth of all births were caesarean sections, ranging from 14.5 to 35.6% in West Bank hospitals and from 16.6 to 26.0% in Gaza hospitals [5]. To appropriately address the rising caesarean section rates, the causes for these large variations between government hospitals need to be understood. Reasons for the wide variation in caesarean section rates across different countries are still unknown, but it has been suggested that social, cultural, unequal access to health services and clinical practice patterns might be major contributing factors [7–9]. Moreover, it has been shown that these variations are mainly due to differences in intrapartum caesarean section rates [10]. A previous study showed that differences in sociodemographic and obstetric characteristics of the population in six study hospitals did not explain variation in intrapartum caesarean section rates in these hospitals [11]. However, indications for intrapartum caesarean section, as one possible reason for varying rates, have not so far been studied in Palestine. Information on the intrapartum caesarean section indications will be useful for physicians and public health providers to assess the practice and improve maternal health outcomes in Palestine [9, 12].

This study aims to investigate variation in odds for intrapartum caesarean section between government hospitals in Palestine and explore whether potential differences can be explained by differences in indications.

## Methods

The data were obtained from The Palestinian Perineum and Birth Complications Study, a prospective cohort study comprising six Palestinian government hospitals. All women scheduled for vaginal delivery, including the referred patients, in the period from 1 March 2015 until 30 November 2016 were included in the study [5].

**Fig. 1:**
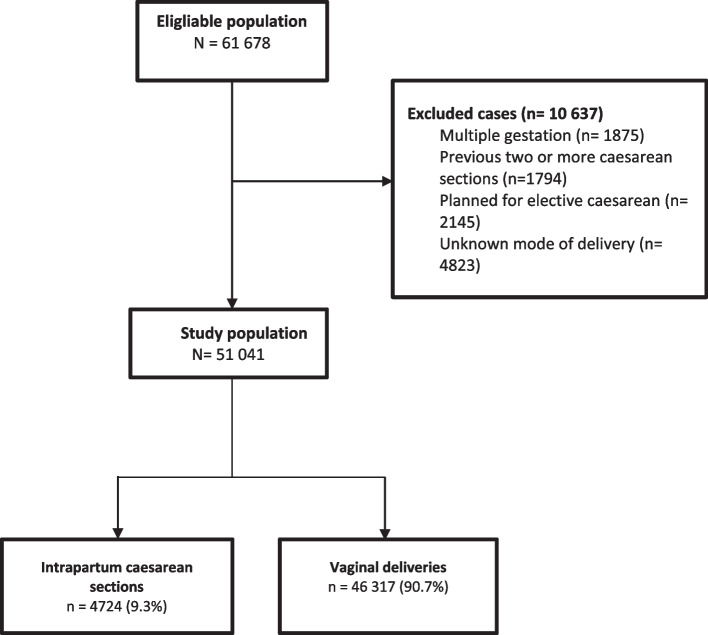
Flow chart of the selected study population, multicenter study from Palestine

Women with multiple gestations, with previous ≥ two caesarean section, those planned for elective caesarean section and women with missing information about mode of delivery were excluded (Fig. 1). Three of the selected hospitals were located in Gaza and three in the West Bank. The hospitals were government teaching as well as referral hospitals, except for Hospital 2 which was not a referral hospital, and Hospital 3 which was not a teaching hospital.

### Data collection and entry

Maternal pre-pregnancy data were collected from the mother and child health handbook while intrapartum data were collected from the medical records. The process of data collection began when the women were admitted for vaginal delivery and followed up until discharge from the hospital.

Data on maternal sociodemographic and obstetric characteristics, mode of delivery and caesarean section indications were collected prospectively using case registration forms [5]. Data on indications for intrapartum caesarean section were registered according to the decision makers (senior doctors), with multiple indications or only one indication. Then all data were entered into special software (DHIS 2) [5].. Data were transferred from DHIS 2 to be saved in the Service for Sensitive Data (TSD) platform. TSD is developed and operated by the University of Oslo for researchers to collect, save, analyse and share sensitive data in compliance with Norwegian regulations regarding individuals’ privacy.

### Risk factors

Sociodemographic characteristics included maternal age, education and pre-pregnancy body mass index (BMI, kg/m^2^). While obstetric characteristics were average number of children alive, history of previous caesarean section and in vitro fertilisation treatment (IVF). Mode of delivery was dichotomised into vaginal delivery and intrapartum caesarean section. The criteria for intrapartum caesarean section in the studied hospitals reflect Lucas urgency classification one, two, or three [13].

Indications for intrapartum caesarean section were grouped into six diagnostic categories: 1- Fetal distress, diagnosed as pathologic findings by cardiotocography and/or meconium stained amniotic fluid. 2- Failure to progress included cephalopelvic disproportion, ineffective contractions, failed forceps/vacuum, maternal exhaustion and fetal malpresentation. 3- Breech presentation. 4- previous caesarean section with an additional obstetric indication such as short interval between the previous caesarean section and the next pregnancy, post term and prelabor rupture of membranes (PROM). 5- Hypertensive disorders included chronic hypertension, gestational hypertension, preeclampsia and eclampsia. 6- Others, included antepartum bleeding and any other indications necessitating intrapartum caesarean section.

### Outcomes

The primary outcome was the intrapartum caesarean section among singleton pregnancies for five Palestinian hospitals as compared to the reference (Hospital 1). The secondary outcome was the commonest indication for intrapartum caesarean section among nulliparous and parous women with singleton pregnancies.

### Statistical analyses

Statistical analysis was performed by using descriptive statistics for the sociodemographic and obstetric characteristics of the women, presented as frequencies and proportions, and as means with standard deviations (SD) by the hospital. Comparison of proportions was tested by χ2 test and differences in means by one-way ANOVA analysis. *P* < 0.05 was considered statistically significant.

Logistic regression was used to estimate the association between hospital, sociodemographic, obstetric characteristics and indications for the odds of intrapartum caesarean section. The strength of association was estimated by crude and adjusted odds ratios (ORs) with 95% confidence intervals (CIs). Hospital 1, which had the highest volume of deliveries, was used as a reference. To investigate whether differences in indications could explain differences in odds of intrapartum caesarean section between hospitals, two models were tested. Model 1 included sociodemographic characteristics (maternal age, education and pre-pregnancy BMI) and obstetric characteristics (average number of children alive, history of previous caesarean section and IVF), previously shown to be associated with intrapartum caesarean section [11], while Model 2 additionally adjusted for indications for intrapartum caesarean section. The amount of explained variance by the model was given by Nagelkerke R square. The difference in R^2^ between model 1 and model 2 was considered as the fraction of the variation in intrapartum caesarean section that can be explained by the indications. Data were analysed in the different strata according to whether the women were nulliparous or parous. No multicollinearity was found among both groups. The reliability of selected variables of data showed the correct data registration and entry to be more than 80% which reflected in Kappas varying from 0.95 to 1.0 [5].. All statistical analyses were performed using SPSS 22 (version 22.0, Chicago, IL, USA).

## Results

Of the total of 61,678 women who planned for vaginal birth during the study period, 10,637 women were excluded (Fig. 1). Among the remaining 51,041 singleton pregnant women, 4724 (9.3%) women had an intrapartum caesarean section.

Table 1 describes the differences in sociodemographic and obstetric characteristics between the study hospitals. The overall mean age for all women in the study was 26.5 years (SD 5.7). The majority of women had 10–12 years of education (Table 1). More than 50% of women had BMI < 25 in all hospitals except in Hospital 1.

**Table 1:** Sociodemographic and obstetric characteristics of the study population (*N =* 51,041)

	Hospital 1 (***N =*** 17,314) ***N*** (%)	Hospital 2 (***N =*** 7557) ***N*** (%)	Hospital 3 (***N =*** 7397) ***N*** (%)	Hospital 4 (***N =*** 7898) ***N*** (%)	Hospital 5 (***N =*** 6152) ***N*** (%)	Hospital 6 (***N =*** 4723) ***N*** (%)	***P*** value
	**Gaza**			**West Bank**			
Age (years)^a^	25.6 ± 5.6	26.8 ± 5.7	27.5 ± 5.7	26.7 ± 6.0	26.6 ± 5.5	26.4 ± 5.5	< 0.001
Education (years)							
≤9	777 (4.5)	764 (10.1)	274 (3.7)	1172 (14.8)	838 (13.6)	1505 (31.9)	< 0.001
10–12	11,229 (64.9)	4724 (62.5)	4118 (55.7)	4413 (55.9)	3032 (49.3)	1817 (38.5)	
≥13	5308 (30.7)	2069 (27.4)	3005 (40.6)	2313 (29.3)	2282 (37.1)	1401 (29.7)	
Missing	0	0	0	0	0	0	
BMI (kg/m^2^)							
≤18.5	204 (1.2)	37 (0.5)	89 (1.3)	424 (5.5)	339 (5.6)	189 (5.4)	< 0.001
18.5–24.9	4511 (26.4)	4012 (59.2)	3473 (48.9)	4525 (58.8)	3635 (60.4)	2245 (63.6)	
25–29.9	9070 (53.1)	2387 (35.2)	2997 (42.2)	2138 (27.8)	1539 (25.6)	888 (25.1)	
≥ 30	3293 (19.3)	341 (5.0)	547 (7.7)	613 (8.0)	510 (8.5)	209 (5.9)	
Missing	236	780	291	198	129	1192	
Nulliparous	5361 (31.0)	1949 (25.8)	1977 (26.7)	1859 (23.5)	1840 (29.9)	1391 (29.5)	< 0.001
Number of children alive^b^							
0–1	3744 (31.3)	1353 (24.1)	1495 (27.6)	1656 (27.4)	1532 (35.5)	1156 (34.7)	< 0.001
≥2	8209 (68.7)	4255 (75.9)	3925 (72.4)	4383 (72.6)	2780 (64.5)	2176 (65.3)	
previous one caesarean section^b^	1312 (11.0)	322 (5.7)	455 (8.4)	869 (14.4)	563 (13.1)	295 (8.9)	
IVF							
	56 (0.3)	45 (0.6)	16 (0.2)	41 (0.5)	36 (0.6)	26 (0.6)	< 0.001

*BMI* body mass index, *IVF* in vitro fertilisation treatment

^a^ Data presented as mean ± SD

^b^ Among parous women

The prevalence of intrapartum caesarean section varied significantly between hospitals (Table 2); from 7.6% in Hospital 2 to 22.1% in Hospital 6 for nulliparous women, and from 5.8% in Hospital 2 to 14.4% in Hospital 6 among parous women.

**Table 2:** Prevalence of intrapartum caesarean section in the study hospitals (*N =* 51,041)

	Hospital 1 ***N*** (%)	Hospital 2 ***N*** (%)	Hospital 3 ***N*** (%)	Hospital 4 ***N*** (%)	Hospital 5 ***N*** (%)	Hospital 6 ***N*** (%)
	**Gaza**			**West Bank**		
Nulliparous	613/5361 (11.4)	149/1949 (7.6)	234/1977 (11.8)	180/1859 (9.7)	256/1840 (13.9)	307/1391 (22.1)
Parous	925/11953 (7.7)	326/5608 (5.8)	420/5420 (7.7)	384/6039 (6.4)	450/4312 (10.4)	480/3332 (14.4)
Total	1538/17314 (8.9)	475/7557 (6.3)	654/7397 (8.8)	564/7898 (7.1)	706/6152 (11.5)	787/4723 (16.7)

Data presented as number of women who had caesarean section in the group/total number of women in the group (percentage)

Indication was given for the majority (88.3%) of the women with intrapartum caesarean section (Table 3). The mean number of indications per woman was 1.21 (5033/4169). The most common indications among nulliparous women were failure to progress in Hospitals 1, 2 and 3 and fetal distress in Hospitals 4, 5 and 6 (Table 3). Hospital 2 had the highest prevalence of hypertensive disorder as an indication at 14.4%. For parous women, the commonest indication in Hospitals 3, 5 and 6 was previous caesarean section with additional obstetric indication, while this was failure to progress in Hospital 1, breech presentation in Hospital 2 and fetal distress in Hospital 4 (Table 3).

**Table 3:** Indications for intrapartum caesarean section in the study hospitals (*N =* 4724)

	Hospital 1 (***N =*** 1538) ***N*** (%)	Hospital 2 (***N =*** 475) ***N*** (%)	Hospital 3 (***N =*** 654) ***N*** (%)	Hospital 4 (***N =*** 564) ***N*** (%)	Hospital 5 (***N =*** 706) ***N*** (%)	Hospital 6 (***N =*** 787) ***N*** (%)
	**Gaza**			**West Bank**		
Nulliparous (*N =* 1739)	(*N =* 613)	(*N =* 149)	(*N =* 234)	(*N =* 180)	(*N =* 256)	(*N =* 307)
Fetal distress	161 (26.3)	10 (6.7)	91 (38.9)	90 (50.0)	74 (28.9)	89 (29.0)
Failure to progress	256 (41.8)	35 (23.5)	92 (39.3)	36 (20.0)	68 (26.6)	74 (24.1)
Breech	83 (13.5)	30 (20.1)	38 (16.2)	41 (22.8)	31 (12.1)	28 (9.1)
Hypertension disorder	30 (4.9)	22 (14.8)	11 (4.7)	9 (5.0)	15 (5.9)	28 (9.1)
Others	118 (19.2)	21 (14.1)	31 (13.2)	10 (5.6)	53 (20.7)	72 (23.5)
Missing	75 (12.2)	37 (24.8)	21 (9.0)	8 (4.4)	54 (21.1)	26 (8.5)
Parous (*N =* 2985)	(*N =* 925)	(*N =* 326)	(*N =* 420)	(*N =* 384)	(*N =* 450)	(*N =* 480)
Fetal distress	224 (24.2)	21 (6.4)	83 (19.8)	151 (39.3)	52 (11.6)	67 (14.0)
Failure to progress	309 (33.4)	64 (19.6)	124 (29.5)	67 (17.4)	95 (21.1)	35 (7.3)
Breech	122 (13.2)	66 (20.2)	80 (19.0)	105 (27.3)	50 (11.1)	69 (14.4)
Previous caesarean section with additional obstetric indication	165 (17.8)	47 (14.4)	152 (36.2)	54 (14.1)	160 (35.6)	204 (42.5)
Hypertension disorder	46 (5.0)	34 (10.4)	28 (6.7)	23 (6.0)	37 (8.2)	27 (5.6)
Others	161 (17.4)	30 (9.2)	68 (16.2)	59 (15.4)	118 (26.2)	89 (18.5)
Missing	94 (10.2)	92 (28.2)	38 (9.0)	7 (1.8)	77 (17.1)	26 (5.4)

Data presented as number (percentage)

The percentage of intrapartum caesarean section equal more than 100% due to 806 women (19.3%) having more than one indication

Table 4 shows crude and adjusted ORs for intrapartum caesarean section stratified by parity. Among nulliparous women the crude ORs for intrapartum caesarean section differed by the hospital. Compared to Hospital 1, the largest difference was found for Hospital 6 and the lowest was found for Hospital 2. Adjustment for sociodemographic and obstetric characteristics slightly influenced the results. Moreover, after additional adjustment for indications, still some differences in odds for intrapartum caesarean section were observed (Table 4, model 2). The amount of explained variance increased from 5.0% in model 1 to 63.3% in model 2, thus indication explained 58.4% of the variability in intrapartum caesarean section.

**Table 4:** Crude and adjusted odds ratios (ORs) with 95% confidence intervals (CIs) of intrapartum caesarean section across study hospitals

Hospitals	Crude OR (95% CI)	Model 1^**a**^ OR (95% CI)	Model 2^**b**^ OR (95% CI)
**Nulliparous**			
Hospital 1	ref.	ref.	ref.
Hospital 2	0.64 (0.53 to 0.77)	0.60 (0.49 to 0.74)	1.12 (0.79 to 1.58)
Hospital 3	1.04 (0.89 to 1.22)	0.88 (0.74 to 1.04)	1.06 (0.77 to 1.45)
Hospital 4	0.83 (0.70 to 0.99)	0.84 (0.69 to 1.02)	0.42 (0.31 to 0.57)
Hospital 5	1.25 (1.07 to 1.46)	1.18 (0.99 to 1.41)	2.18 (1.61 to 2.96)
Hospital 6	2.19 (1.88 to 2.55)	1.88 (1.54 to 2.28)	2.41 (1.70 to 3.40)
**Parous**			
Hospital 1	ref.	ref.	ref.
Hospital 2	0.74 (0.65 to 0.84)	0.73 (0.64 to 0.85)	1.94 (1.51 to 2.50)
Hospital 3	1.00 (0.89 to 1.13)	0.94 (0.83 to 1.06)	0.90 (0.70 to 1.16)
Hospital 4	0.81 (0.72 to 0.92)	0.78 (0.68 to 0.88)	0.50 (0.40 to 0.63)
Hospital 5	1.39 (1.23 to 1.56)	1.35 (1.19 to 1.53)	2.07 (1.61 to 2.67)
Hospital 6	2.01 (1.78 to 2.26)	1.80 (1.56 to 2.08)	1.77 (1.33 to 2.35)

^a^ Adjusted for sociodemographic (maternal age, education and pre-pregnancy body mass index) and obstetric characteristics (average number of children alive, history of previous caesarean section and in vitro fertilization treatment)

^c^ Adjusted for sociodemographic (maternal age, education and pre-pregnancy body mass index) and obstetric characteristics (average number of children alive, history of previous caesarean section and in vitro fertilisation treatment) and intrapartum caesarean section indications (Fetal distress, failure to progress, breech, previous caesarean section with additional obstetric indication, hypertension disorder and others)

Among parous women, the crude ORs were similar to that of nulliparous women. Adjustment for sociodemographic and obstetric characteristics influenced the ORs for all hospitals only to a small degree. When indication was included in the model, the OR of intrapartum caesarean section almost doubled in Hospitals 2, 5 and 6 compared to Hospital 1 and nearly halved in Hospital 4 while no difference was observed for Hospital 3 (table 4). Among parous women the amount of explained variance attributed to indications was 66.4% (Nagelkerke R^2^ increased from 2.0% in model 1 to 68.4% in model 2).

## Discussion

Large variations in intrapartum caesarean section rates were observed between hospitals among singleton pregnancies both in nulliparous and parous women. The differences in odds for intrapartum caesarean section could not be fully explained by differences in indications, although for nulliparous women 58% of the variability in intrapartum caesarean section could be explained by variation in indications; the corresponding percentage for parous women was 66%, respectively.

As the study hospitals were public government, they had similar work environments and available tools. However, the rates of intrapartum caesarean section varied significantly between the study hospitals with the lowest rate in Hospital 2 and the highest in Hospital 6. Hospital 2, not a referral hospital, transferred the high-risk patients to hospitals with intensive care facilities. This factor may contribute to its intrapartum caesarean section rate to be the lowest. The high caesarean section rate in Hospital 6 could not be explained by maternal factors, therefore, obstetric practice and decision makers may play an important role [14, 15]. Variations in staff working schedules, clinical experience and level of knowledge of those who decide to conduct caesarean section may also contribute to explain the differences in risks for intrapartum caesarean section between the study hospitals [16].

In concordance with previous studies [17], the most common reasons for caesarean section among nulliparous women in this study were fetal distress and failure to progress with wide variations between hospitals. Electronic fetal monitoring, which was routinely used in the study hospitals, is associated with an increased likelihood of caesarean section [16]. Furthermore, the lack of fetal scalp sampling might cause over-diagnosis [18]. Moreover, non-judicious use of oxytocin augmentation to manage large numbers of deliveries might increase the risk of fetal distress [19].

Previous caesarean section with additional obstetric indication was the commonest indication among parous women with large variations between hospitals. The fear of litigation related to uterine rupture and associated risks to the mother and the fetus, might explain some variations [20, 21]. In Palestine, no medico-legal framework or indemnity for doctors exists in case of maternal or fetal complications occurring during obstetric care and procedures. Moreover, increased awareness of potential complications of vaginal delivery resulted in obstetricians having a lower threshold for advising delivery by caesarean section especially when accompanied with an additional obstetric indication [22].

The indications influenced the odds of intrapartum caesarean section differently in each study hospital. Among nulliparous women fetal distress increased the odds of intrapartum caesarean section to a larger extent in Hospital 3 than in the remaining hospitals. Among parous women, fetal distress increased the odds of intrapartum caesarean section to a larger extent in Hospitals 1, 3 and 4 than in Hospitals 2, 5 and 6. This may demonstrate a wide range in obstetric care practice between the hospitals as well as wide variations in physicians’ subjective diagnosis that make the distribution of the commonest indications vary between hospitals [9, 10, 17, 23–25]. Therefore some variations might be due to varying hospital culture emphasizing on different indications [23, 24], which became apparent when some hospitals, such as Hospital 6, mainly had one indication per woman, whereas others, such as Hospital 5, reported multiple indications in a larger proportion of women. Furthermore, physicians’ may differ in their choice of indication, when multiple indications may apply, reflecting differing clinical practices rather than differing medical situations [9]. Accordingly, similar trends were observed in two study hospitals located in the Gaza-Strip, and may reflect shared beliefs and work environments. Interestingly, in the hospital with the highest intrapartum caesarean section rate, indications did not influence the rate, suggesting an overall lower threshold for decision towards intrapartum caesarean section irrespective of indication.

Several studies have reported significant variation in caesarean section rates between hospitals. Gillian studied rates of primary caesarean section in 16 health service delivery areas in British Columbia and found caesarean section rates ranging from 16.1 to 27.5% between areas [24]. This variation could not be explained by patient illness or indications of caesarean section, but reflected differing medical decision making. However, these results contrast those from a study in Nova Scotia, which explained high caesarean section rates by maternal characteristics [26].

Another large study from England, comparing 146 National Health Service trusts, showed large variation in rates of intrapartum caesarean section singleton pregnancies in different trusts [10]. Likewise, two studies from the USA showed wide variations in caesarean section rates among different facilities [8, 27]. The authors suggested that these variations were due to lack of precise criteria for indications. Our study showed similar findings which may suggest lack of guidance for clinical decision making across the study hospitals, and implies a wide range in obstetric care practice patterns and work culture [27]. The recently updated Palestinian national guidelines for standardised labour management may contribute to harmonise clinical practice [28].

Therefore, reduction of hospital variations in caesarean section prevalence and indications is essential and has to be achieved by a multimodal approach including continuous staff training and increased instrumental deliveries among low-risk groups. One further aspect is to increase evidence-based practice among Palestinian obstetricians and midwives, as lack of such might be one of the reasons for the variations in frequency of common indications. Furthermore, this study as well as ongoing local audits might have practical implications for health service planners to focus on the commonest caesarean section indications and the decision makers in order to standardize maternity care and improve quality of care and maternal health outcomes.

### Strengths and limitations

The data were collected for research purposes in a prospective manner. All women aiming to give birth vaginally during the study period were included, reducing the risk for selection bias. Also, indications for intrapartum caesarean section were registered by attending medical teams and thus reducing time related bias.

The main limitation of this study was the missing data, where almost 10% of the potential population was excluded because of missing information on mode of delivery as well as missing values on indications. The missing values were considered to be random and should therefore not influence the effect estimates. Hassan S et al. tested the validity of data and found that data is considered reliable for research purposes [5]. Additionally, the data did not contain specific definitions or details about diagnostic criteria for registered indications. Some of the studied indications were diagnosed subjectively depending on decision makers, with some women having more than one indication. This may affect prioritisation of the prime indication to varying degrees in different hospitals and by different decision makers. This study did not include private hospitals, a because most deliveries in Palestine take place in the government hospitals which was our main focus.

## Conclusion

Large differences in rates for intrapartum caesarean section were observed between the six government Palestinian hospitals. These could not be explained by differences in the indications for intrapartum caesarean section, suggesting additional factors may influence clinical practice. These findings may imply that a wide range in obstetric care practice patterns, different strategies and varying work culture played an important role in the decision to deliver by intrapartum caesarean section. A need for change exists in the healthcare system with greater emphasis on resources, education, continuing professional development and clinical governance. Further investigation on provider related factors as well as maternal and fetal outcomes in different hospitals, is necessary.

## Data Availability

The dataset used in this audit is available on reasonable request from the first author’s (MWZ), but cannot be publicly shared to maintain women’s confidentiality.

## Abbreviations

CEmONC: Comprehensive emergency obstetric and neonatal care
WHO: World Health Organization
IVF: in vitro fertilisation treatment
TSD: Service for Sensitive Data
DHIS2: District Health Information Software 2
BMI: Body mass index
SD: Standard deviation
OR: Odds ratios
CI: Confidence interval
